# Forty Years Abuse of Baking Soda, Rhabdomyolysis, Glomerulonephritis, Hypertension Leading to Renal Failure: A Case Report

**DOI:** 10.4137/ccrep.s888

**Published:** 2008-06-17

**Authors:** Terje Forslund, Arvo Koistinen, Jorma Anttinen, Bodo Wagner, Marja Miettinen

**Affiliations:** 1 Division of Nephrology, Department of Internal Medicine, Jyväskylä, Finland.; 2 Department of Pathology, Jyväskylä, Finland.; 3 Department of Anesthesiology and Intensive Care Central Finland Health Care District Hospital, Jyväskylä, Finland.

**Keywords:** baking soda intoxication, rhabdomyolysis, glomerulonephritis, hypertension, end-stage renal failure

## Abstract

We present a patient who had ingested sodium bicarbonate for treatment of alcoholic dyspepsia during forty years at increasing doses. During the last year he had used more than 50 grams daily. He presented with metabolic alkalosis, epileptic convulsions, subdural hematoma, hypertension and rhabdomyolysis with end stage renal failure, for which he had to be given regular intermittent hemodialysis treatment. Untreated hypertension and glomerulonephritis was probably present prior to all these acute incidents. Examination of the kidney biopsy revealed mesangial proliferative glomerulonephritis and arterial wall thickening causing nephrosclerosis together with interstitial calcinosis. The combination of all these pathologic changes might be responsible for the development of progressive chronic renal failure ending up with the need for continuous intermittent hemodialysis treatment.

## Introduction

In the 1800 century baking soda (BS; NaHCO_3_) was used to put air into breads and cakes, and it was a common ingredient in many baking goods to cause batter and dough to rise. BS was also a common home remedy to treat dyspepsia. Intake of large amounts of BS could cause serious adverse effects with hypokalemic hypochloremia, metabolic alkalosis, hypernatremia, and hypocalcaemia (1,2). Prolonged administration of NaHCO_3_ (NaBic) did not produce renal failure (RF) unless volume depletion was present (1). Resistant hypertension (3), cardiopulmonary arrest (4), and spontaneous rupture of the stomach with lethal outcome due to BS ingestion have been described (5,6). Short-time of high-dose BS intake may cause acute intoxications (7,8) and unintentional or accidental intake in children (9,10,11) have been reported. Pica, often unrecognized, is a well known habit in pregnant women and in dialysis patients. Life-threatening BS pica has been reported in a hemodialysis (HD) patient (12) and in a pregnant woman who developed rhabdomyolysis (13). Long-term abuse of BS is rare and periods of its intake more than 8 years (14) was so far not reported.

Here we report a subject who ingested BS at increasing doses during a period of forty years resulting in life-threatening complications with metabolic alkalosis, epileptiformic convulsions, subdural hemorrhage, and rhabdomyolysis. Kidney biopsy showed mesangial and interstitial changes with calcifications pointing to previous glomerulonephritis. Chronic renal failure developed and regular intermittent hemodialysis treatment had to be given.

## Case

A sixty-four year old man, with no previous medication and no previous visit to any doctor or nurse, was sent to our emergency department due acute epileptiformic convulsions. He was working at an auto-repair-shop where one morning his colleagues observed that he was confused and he fell from his chair with convulsions. At arrival to the hospital he was unconscious and he appeared hypovolemic and dehydrated. Pupils, thus moving around had normal reactions to light and no paretic regions were registered. Neurological reflexes were normal and symmetric, and muscles of the extremities were slightly rigid.

He had a history of many years of alcohol abuse which included short episodes of extreme drinking. At arrival to the hospital he tested negative for alcohol, barbiturates and benzodiazepine. Initially, the blood pressure (BP) had been measured to 195/148 mmHg and had fallen to 130/104 mmHg at arrival. Heart rate was 100 beats/min, regular, with normal heart and lung status. Electrocardiogram showed slightly descending ST levels in lateral leads. Chest x-ray was normal. Fever was absent. Plasma creatinine (p-creat) and urea nitrogen (p-urea) concentration was 780 μmol/l and 33 mmol/l, respectively. Serum creatinine kinase (CK) concentration was 2980 U/L (ref. 40–280 U/L) decreasing to 309 U/L during the next 4 days. The pro-brain natriuretic peptide (pro-BNP) concentration was 28100 ng/l (ref. < 194 ng/l). Blood glycosylated hemoglobin fraction was 5.2% (ref. 4–6%). Central venous pressure was 0–1 mm Hg. No signs of bleeding was found and INR-test was normal (<1.0). Arterial blood oxygen saturation was 95–98% without additional external oxygen supply. Metabolic parameters were heavily pathologic showing severe alkalosis and hypochloremia (Table 1).

Computed tomography (CT) scan examination of the brain disclosed a 4 mm thick subdural hemorrhage in the frontal-temporal-parietal region, which was considered not to need surgical intervention. Repeat CT scan six days later demonstrated shrinking of the hematoma to 2 mm of thickness. BP and tachycardia was controlled with multiple i.v. doses (2.5 to 5 mg) of metoprolol tartrate (Seloken®, Astra-Zeneca, Sweden) followed by orally given metoprolol succinate (Seloken®, Astra-Zeneca, Sweden), lercandipine hydrochloride (Zanidip®, Leiras, Finland), and moxonidine (Physiotens®, Solvay Pharma, Finland). Later, the BP was kept at 120/70 mmHg with 5 mg of bisoprolol hemifumarate (Emconcor®, Merck) alone. In spite of a daily diuresis of 2650 ml p-creat and p-urea concentrations remained elevated why regular intermittent hemodialysis (HD) had to be initiated. Some weeks later the urine excretion diminished to >200 ml/day. Kidney ultrasound examination revealed normal sized kidneys and one month after admission a kidney biopsy was taken.

After correction of the metabolic alkalosis, the patient regained consciousness and could give an exact history of his alcohol and BS abuse. He had eaten BS during 40 years due to dyspepsia caused by alcohol consumption. Initially he took 10–15 grams daily, slowly increasing the amount to about 30–35 grams daily, and during the last year he ingested more than 50 gram of BS every day (about 1.5–2.0 kg/month). He was a heavy tobacco smoker. He had a little amount of proteinuria (171 mg/24 hrs) with no hematuria (after urinary bladder catheter removal). Anti-neutrophil cytoplasmic antibodies and anti-basement membrane antibodies were absent. Serum protein fraction was normal with normal IgA, IgM and IgG levels.

### Kidney biopsy

**Figure 1:**
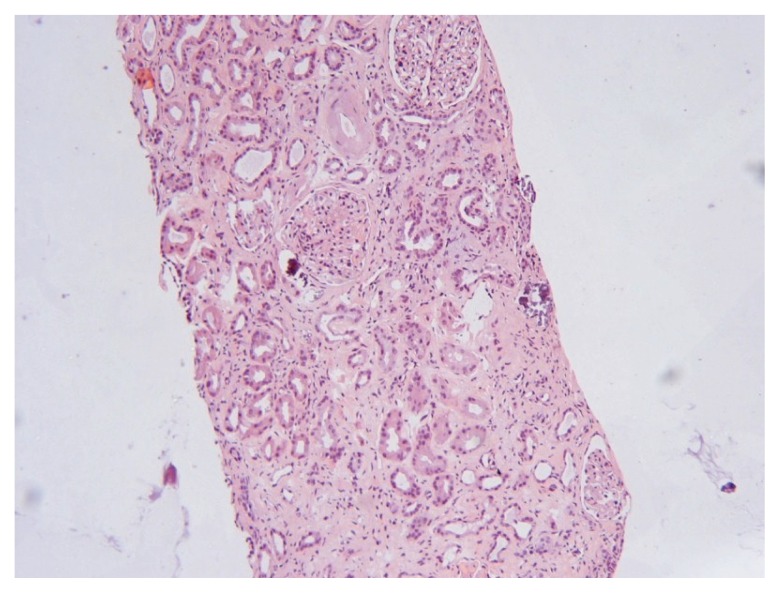
Overview of the renal biopsy showing slight increase of mesangial material and interstitial calcium deposits (HE stain, magnification 125x).

**Figure 2:**
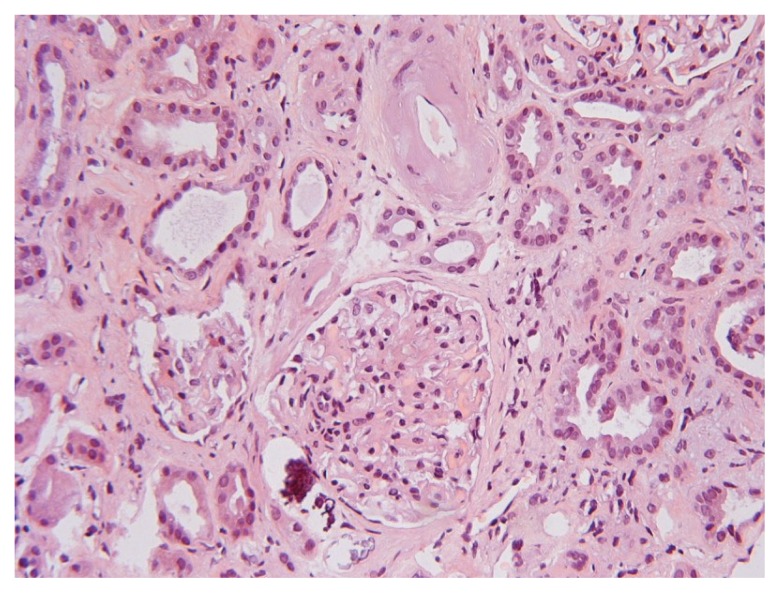
Glomeruli with increased mesangial material and calcification outside glomerular capsule (HE stain, magnification 250x).

The biopsy specimen contained 9 glomeruli of which 2 were totally sclerosed (Fig. 1). Glomerular mesangial matrix mass was slightly increased with no hypercellularity, the capillaries were open, and the glomerular basement membrane was intact at silver stain. Hyaline sclerosis was observed in the wall of small pre-glomerular arterioles (Fig. 2). Areas of fibrosis and calcifications together with lymphocytic infiltration were observed in the interstitium (Figs. 1 and 2). The kidney biopsy disclosed a low grade mesangial proliferative glomerulonephritis (GN) combined with arteriolar hyaline sclerosis and metastatic interstitial fibrosis and calcifications interpreted as hypertensive nephrosclerosis. Immunofluorescence was negative and neither complement nor fibrin was found. Examination with electron microscopy was not performed.

### Gastroscopy

A gastroscopy examination was performed 6 days after admission to our hospital and disclosed mild eosophagitis with some initial fields of venous dilatation with no bleeding, no varicose veins or Barrett-like deformities. The pyloric region was extremely deformed pointing to plural recurrent duodenal ulcers. The ventricle had hypertensive gastropathic deformations. The test for Helicobacter bacteria was negative.

## Discussion

This is the first report on a subject who abused BS during forty years and more than 50 gr. daily during the last year. Like in previously reports (1,2), the BS ingestion produced severe metabolic alkalosis and electrolytic imbalance, complicated with epileptic convulsions and rhabdomyolysis, subdural hematoma leading to hospitalization and treatment in the intensive care unit with continuous, and later intermittent hemodialysis treatment. The metabolic derangements due to chronic bicarbonate ingestion may cause sustained hypokalemia, hypernatremia, and hypoxia, with variable clinical presentations including seizures (15). The acute seizure assumable caused that the patient fell directly to the floor from the chair, hitting the head, which most probably caused the subdural hemorrhage as a secondary event. Whether the initial measured high blood pressure (195/148 mmHg, see above) contributed to development of the subdural hemorrhage or secondary caused by the hemorrhage remains an unanswered question. Beside chronic alcohol abuse and its abrupt withdrawal, seizures, hypoglycemia, and hypokalemia, may all together have contributed to rhabdomyolysis (16). Moreover, co-existent hypophosphatemia and hypomagnesiumemia, although here not mentioned, might in theory have participated in this development (17,18).

The previous undiscovered mesangial proliferative glomerulonephritis (GN), untreated hypertension and nephrosclerosis had probably been present prior to this episode. We suggest that the BS abuse blunted the metabolic acidosis that most often develops during progressive chronic renal failure which also explains why clinical symptoms did not manifest earlier in the course. Although renal failure was provoked by the dehydration, rhabdomyolysis, and metabolic disturbances, the GN most probably contributed to the deterioration of kidney function (18). Absence of pigment nephropathy and no signs of acute tubular necrosis in the kidney biopsy also point more to a previous longstanding GN with hypertension than acute rhabdomyolysis as reason for renal failure. It also seemed unlikely that BS alone, even when ingested at such high amounts, could cause GN as demonstrated here. The interstitial calcification found at kidney biopsy may be interpreted as caused by BS misuse together with secondary hyperparathyroidism since the parathyroid hormone level was increased (Table 1), thus such cases have been reported in hemodialysis patients (19). To our knowledge, descriptions of kidney biopsies from patients with renal failure and long-term BS abuse have not been described in the literature.

Acute infusion of bicarbonate in animal experiments (20) and long-term ingestion of NaBic in man (1) had no significant effect on renal function if not simultaneously being dehydrated. In addition, our patient had rhabdomyolysis, a well known reason for developing acute renal failure, which is mostly reversible when appropriate treated. Moreover, alcohol abuse may cause volume contraction and chronic alcoholic users represent a particular population at risk (15). During alcohol intake the dehydration could in part be explained by increased diuresis due to loss of vasopressin function (21). The period of time during which dehydration had been present prior to the events in our patient was not known.

In the two cases reported by Fitzgibbons and Snoey (15) the duration of BS ingestion was not mentioned although one might assume that those patients had used BS over a very long period of time (15). Progressive renal failure and decreased glomerular filtration rate may reduce the filtered load of bicarbonate thus aggravating the metabolic alkalosis. While intake of bicarbonate during 3 weeks with daily doses up to 140 g did not notably influence renal function (1), it is a huge difference between three weeks of BS intake (1) and increasing amounts of BS during forty years.

A temporary rise in serum creatinine concentration has been reported, which at a second occasion of BS intoxication remained at a high level (15), not requiring hemodialysis treatment, however. No histology was reported in that case (15), and the renal failure was probably brought about by a pre-existing renal disease or other causes and not exclusively due to BS intoxication. Although mesangial proliferative GN was found, the histological changes were mild (Figs. 1 and 2) and a clear discrepancy between these rather mild changes and the degree of renal failure needing continuous intermittent hemodialysis treatment existed, for which we have no consistent explanation. The decision to initiate hemodialysis treatment was made upon the fact that a decline in urine production together with an increase in p-creat and a constantly high p-urea-nitrogen concentration was observed within the first week of medical treatment (Table 1).

BS pica-associated rhabdomyolysis may result in temporary elevation of the creatine phosphokinase (CK) concentration to 32.750 U/L with no rise in serum creatinine concentration (13). Our patient had a less severe rhabdomyolysis as demonstrated by much lower CK values, and it might be disputed whether rhabdomyolysis contributed to the renal failure in our case. Rhabdomyolysis is often treated with bicarbonate infusion (22) and long-term use of BS might well have masked the initial development of rhabdomyolysis.

We assume that the sequence of events was as follows; the presence of a long-standing untreated hypertension and a previous not diagnosed mesangial proliferative glomerulo-nephritis which caused the initial renal failure. Then long-term alcohol and BS abuse provoked convulsions and subsequent rhabdomyolysis associated with severe volume contraction aggravating the renal failure. Concomitantly he had developed a grave metabolic alkalosis with massive disarrangements of the electrolytes contributing to the manifest renal failure. We suggest that the renal failure was not brought about by BS alone in our patient, but it might have participated or provoked its development.

## Figures and Tables

**Table 1:** Some laboratory tests at arrival (1), after 3 days (2), after 7 days (3), and 1½ month later (4).

Laboratory test (ref. value)	1	2	3	4
P-Sodium (137–145 mmol/l)	147	140	139	140
P-Potassium (3.3–4.9 mmol/l)	2.3	4.0	5.0	4.5
S-Chloride (96–111 mmol/l)	46	84	96	NE
P-Creatinine (60–100 μmol/l)	648	753	791	500
fP-Urea (3.5–8.1 mmol/l)	33	30	30	20
fP-PTH-int (10–60 ng/l)	916	–	–	101
P-Ca- ion (1.15–1.3 mmol/l)	0.75	1.07	1.14	1.12
P-CK (40–280 U/L)	2980	504	309	–
P-CRP (<10 mg/l)	14	47	26	4
B-Hgb (134–167 g/l)	123	94	117	97
aB-pH (7.35–7.43)	*7.57	*7.46	c.7.40	–
aB-pO_2_ (10–14 kPa)	*17.8	*8.7	c.6.1	–
aB-pCO_2_ (4.5–6 kPa)	*13.3	*12.2	c.5.2	–
aB-SBC (22–26 mmol/l)	*85.0	*43.2	c.23.8	–
aB-BE (−2.5–+2.5 mmol/l)	*49.6	*19.0	c.−0.4	–
aB-O_2_ Sat (95%–98%)	*97	*96.8	c.89.4	–

**Abbreviations:** P: plasma; S: serum; PTH-int: intact parathyroid hormone, Ca-ion: Calcium ionized at pH 7.4; CK: creatine kinase concentration; CRP: C-reactive protein concentration; Hgb: hemoglobin; aB: arterial blood.

* value with 30% oxygen supply; c. = capillary blood, no oxygen supply given.
